# Continuous On-Chip Cell Washing Using Viscoelastic Microfluidics

**DOI:** 10.3390/mi14091658

**Published:** 2023-08-25

**Authors:** Hyunjung Lim, Minji Kim, Yeongmu Kim, Seunghee Choo, Tae Eun Kim, Jaesung Han, Byoung Joe Han, Chae Seung Lim, Jeonghun Nam

**Affiliations:** 1 Interdisciplinary Program in Precision Public Health (PPH), Korea University, Seoul 02841, Republic of Korea; hyunjunglim.email@gmail.com; 2 Department of AI Electrical and Electronic Engineering, Incheon Jaeneung University, Incheon 22573, Republic of Korea; minjizzang73@gmail.com; 3 Artificial Intelligence (AI)-Bio Research Center, Incheon Jaeneung University, Incheon 21987, Republic of Korea; 4 College of Life Sciences and Bio Engineering, Incheon National University, Incheon 22012, Republic of Korea; 5 Department of Mechanical and Control Technologies, Seoul Cyber University, Seoul 01133, Republic of Korea; 6 Department of Digital Biotech, Incheon Jaeneung University, Incheon 22573, Republic of Korea; 7 Department of Laboratory Medicine, College of Medicine, Korea University, Seoul 08307, Republic of Korea

**Keywords:** white blood cell, washing, co-flow, viscoelastic fluid

## Abstract

Medium exchange of particles/cells to a clean buffer with a low background is essential for biological, chemical, and clinical research, which has been conventionally conducted using centrifugation. However, owing to critical limitations, such as possible cell loss and physical stimulation of cells, microfluidic techniques have been adopted for medium exchange. This study demonstrates a continuous on-chip washing process in a co-flow system using viscoelastic and Newtonian fluids. The co-flow system was constructed by adding a small amount of biocompatible polymer (xanthan gum, XG) to a sample containing particles or cells and introducing Newtonian fluids as sheath flows. Polymer concentration-dependent and particle size-dependent lateral migration of particles in the co-flow system were examined, and then the optimal concentration and the critical particle size for medium exchange were determined at the fixed total flow rate of 100 μL/min. For clinical applications, the continuous on-chip washing of white blood cells (WBCs) in lysed blood samples was demonstrated, and the washing performance was evaluated using a scanning spectrophotometer.

## 1. Introduction

Medium exchange of cells that are initially suspended in a medium with a high background to another buffer with a low background is indispensable for biological and clinical research [1,2]. The process of transferring cells across disparate solutions enhances the sensitivity and accuracy of the analyses because cell sample preparation commonly requires multiple medium exchange steps for chemical reactions, labeling, and washing. Previous studies on solid-phase chemical reactions for cellular or molecular analyses have reported that the mixing of solutions containing particles inevitably increased the distributions in the analysis readout and the dead time of the reactions [3]. A typical example of cell washing is the extraction of white blood cells (WBCs) from lysed whole blood, since long-term exposure of WBCs to lysis buffer is detrimental and cell lysates can be background noise for post-analyses. Conventional washing of WBCs involves multiple steps such as mixing lysis buffer, centrifugation, removal of cell lysates, and retrieval of WBCs.

Centrifugation is the conventional method for cell washing; however, it has drawbacks such as the use of expensive and bulky equipment, long time consumption, labor intensiveness, and possible cell loss due to the additional pipetting process, which also varies with operator skill. In addition, the high shear stress induced by the centrifugal force can damage cells. Moreover, throughput is limited because centrifugation involves batch processing and is discontinuous.

According to recent advancements in microfluidics, this method can be used as an alternative to centrifugation to address the aforementioned limitations. Among microfluidic techniques, active methods relying on external force fields have been widely used because of the rapid and uniform medium exchange between solutions, which includes dielectrophoretic [4,5], acoustophoretic [6,7,8,9], and magnetic forces [10,11]. Meanwhile, passive methods utilizing channel geometry and/or the hydrodynamic effects of flow-negating external force fields have also been adopted for medium exchange [12,13,14,15]. More recently, viscoelastic non-Newtonian microfluidics has gained considerable attention because of its intrinsic nonlinear elastic forces, which enable particle/cell manipulation in a relatively simple straight microchannel compared with other passive methods using Newtonian fluids [16,17]. Although inertia-based cell washing can be achieved in a straight channel, the flow rate range and cell size required for efficient washing are limited. Owing to the advantages of viscoelastic microfluidics, it has been applied to not only particle/cell focusing and separation [17,18,19,20,21,22,23,24], but also particle/cell washing [24,25,26].

In the previous cell washing using the co-flow configuration of viscoelastic and Newtonian fluids, Ha et al. implemented microparticle transfer across laminar streams using λ-DNA solution and Newtonian fluid [25], whereas Yuan et al. used poly(ethylene oxide) (PEO) for microparticle/cell washing [26]. λ-DNA has strong viscoelasticity even at extremely low concentrations because of the long relaxation time of DNA molecules [27,28]. However, it is relatively expensive compared to other viscoelastic non-Newtonian fluids. In contrast, PEO solutions have medium elasticity and weak shear thinning and have been most widely used in microfluidic particle/cell manipulation.

In this study, we propose continuous on-chip washing of white blood cells in lysed blood using a co-flow system of viscoelastic non-Newtonian and Newtonian fluids. Here, instead of using λ-DNA and PEO solutions as viscoelastic fluids, we investigated the lateral transfer of cells from a xanthan gum (XG) solution to a Newtonian fluid. XG, as a high-molecular-weight polysaccharide, has been known as being biocompatible and non-toxic, which can be employed in the food industry, medical field, and tissue engineering [29,30,31,32,33]. Recently, the size-dependent lateral migration and separation of microparticles in an XG solution in a straight microchannel have been conducted [34,35,36]. Unlike λ-DNA and PEO solutions, the XG solution has strong shear thinning with little viscoelasticity; therefore, the equilibrium positions of particles in the XG solution are different from those in other viscoelastic solutions. Then, the cell suspension in the XG solution can be introduced along the channel center with minimal shear stress, which can be a strong advantage when dealing with shear-sensitive cells [37]. In the present study, the migration characteristics of particles with different blockage ratios were studied using a co-flow system of XG solutions at various concentrations and Newtonian fluids, which has not yet been examined. Continuous on-chip washing of WBCs in lysed blood samples was demonstrated, and the cell washing performance was evaluated using a spectrophotometer and a hemocytometer.

## 2. Materials and Methods

### 2.1. Device Fabrication

A microfluidic device was fabricated from polydimethylsiloxane (PDMS) using a soft lithography technique with an SU-8 replica mold patterned onto a silicon wafer. The PDMS base and curing agent (Sylgard 184, Dow Corning, Midland, MI, USA) were mixed at a 10:1 ratio, degassed in a vacuum chamber, thermally cured in an oven for 1 h at 80 °C, peeled off from the mold, and bonded onto a glass slide with oxygen plasma (CUTE, Femto Science, Hwaseong, Republic of Korea). The fabricated PDMS microchannel consisted of two inlets, two outlets, and a straight rectangular channel with 100 μm width (*W*), 50 μm height (*H*), and 30 mm length.

### 2.2. Sample Preparation

As a viscoelastic non-Newtonian fluid, XG (X0048, Tokyo Chemical Industry Co., Ltd., Chuo, Tokyo, Japan) was prepared in phosphate-buffered saline (PBS) at concentrations of 50, 100, 250, and 500 ppm to evaluate the effect of viscoelasticity on the medium exchange of particles/cells. To estimate the flow characteristics of particles in XG solution, fluorescent polystyrene particles with diameters of 500 nm, 2, 5, 10, and 13 μm (ThermoFisher, Waltham, MA, USA) were used. The final concentration of the particles suspended in the XG solution was approximately $1\times 10^{5}$ particles/mL.

The rheological properties of XG solutions were measured using a rheometer (AR2000, TA Instruments, New Castle, DE, USA) over a wide range of shear rates at room temperature. The measured viscosities and relaxation times of 50, 100, 250, and 500 ppm XG solutions are summarized in Table 1.

Single-donor human whole blood (Innovative Research, Inc., Novi, MI, USA) was used in this study. Whole blood (1 mL) was mixed with 7 mL of 1× BD FACS lysing solution (BD Biosciences, San Jose, CA, USA), 1 mL of 1× SYBR Green for fluorescent staining of WBCs, and 1 mL of 1000 ppm XG solution containing 500 nm fluorescent particles for visualization of the viscoelastic fluid flow in the co-flow system. The final XG solution concentration was 100 ppm.

### 2.3. Experimental Procedure and Post Analysis

The sample and sheath fluid flow rates of the co-flow system were controlled using a syringe pump (Fusion-4000; Chemyx, Stafford, TX, USA). During the experiments, an inverted microscope (IX71, Olympus, Tokyo, Japan) equipped with a color CCD camera (CS230B, Olympus, Tokyo, Japan) was used to monitor the flow of the particles/cells.

To evaluate the washing performance of the co-flow system, samples injected at the two inlets and collected from the two outlets were examined using a UV/VIS spectrophotometer (Multiskan SkyHigh, ThermoFisher Scientific, Waltham, MA, USA). In addition, for a quantitative evaluation of WBC washing, the recovery rate of WBCs was analyzed based on manual counting using a hemocytometer. This was defined as the ratio of the number of particles collected at outlet B to the total number of WBCs in the sample collected from both outlets.

## 3. Results and Discussion

### 3.1. Working Principle

**Figure 1:**
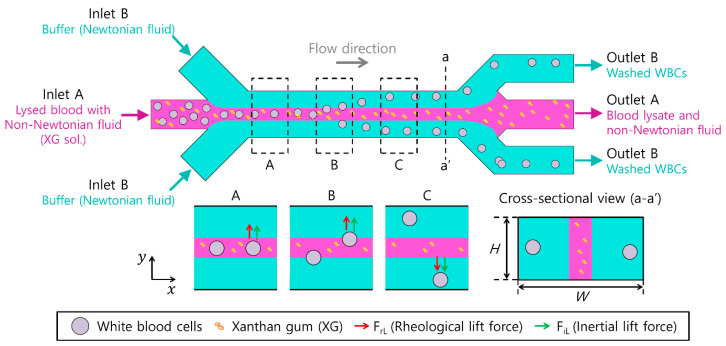
Schematic of continuous on-chip particle/cell washing using a co-flow system of a viscoelastic non-Newtonian and a Newtonian fluid. For WBC washing, sample mixtures containing lysed blood with a non-Newtonian fluid (xanthan gum solution, XG sol.) were introduced to the inlet A, accompanied by sheath fluid (Newtonian fluid) at the inlet B. Due to medium exchange of cells at the Newtonian/non-Newtonian fluid boundary, WBCs were migrated to a clear buffer solution, while blood lysates and non-Newtonian fluid were removed at outlet A.

Figure 1 shows a schematic of continuous on-chip cell washing using the co-flow of a viscoelastic non-Newtonian and a Newtonian fluid. For cell washing, a microchannel with a low aspect ratio (*AR*, AR=H/W, *H* is the channel height and *W* is the channel width) was used, as shown in the cross-sectional view (a-a’) in Figure 1. Cells were injected at the center inlet (inlet B) as focused at the center of the microchannel, with sheath flows of Newtonian fluid injected at the side inlet (inlet A), as shown in Figure 1A.

In a co-flow system, the lateral migration of cells is affected by the rheological lift (*F_rL_*) of strongly shear-thinning and weakly elastic XG solutions [36].

(1) $$F_{rL}\sim a^{3}\nabla N_{1}$$

Here, *a* is the particle diameter, and *N*_1_ is the first normal stress difference. The strong shear thinning of XG solutions tends to drive particle migration to low-shear-rate regions [31]. However, fluid inertial lift (*F_iL_*) also affects cell migration.

(2) $$F_{iL}=F_{iL,w}+F_{iL,s}\sim \rho {\left(a/W\right)}^{4}Q^{2}$$

where ρ is the fluid density, W is the microchannel width, and Q is the total flow rate. $F_{iL,w}$ and $F_{iL,s}$ indicate the wall-induced and shear-gradient-induced inertial lifts, respectively. $F_{iL,w}$ pushes particles/cells away from the channel walls, whereas $F_{iL,s}$ pushes particles/cells to the center of each microchannel face [38]. Based on the synergistic effect of both forces during flow, the flow characteristics of the particles were different for different non-Newtonian fluids. In a viscoelastic non-shear-thinning solution (polyvinylpyrrolidone, PVP) [39], particles migrate towards the center of the microchannel, whereas large particles migrate to two off-centered equilibrium positions, with smaller particles flowing around the centerline in a viscoelastic and weakly shear-thinning fluid (i.e., PEO) [40]. Unlike the other above-mentioned non-Newtonian fluids, the equilibrium positions of the particles/cells in the XG solution were found to be different [36], which has not been studied in co-flow systems with Newtonian fluids.

Owing to the simultaneous effect of inertial and rheological forces in viscoelastic fluid flow, non-dimensional numbers are required to characterize the flow in a microchannel, including the Reynolds number (*Re*), Weissenberg number (*Wi*), and elasticity number (*El*).

(3) $$Re=\frac{\rho V_{m}D_{h}}{\eta }$$

(4) $$Wi=\lambda {\dot{\gamma }}_{c}$$

(5) $$El=\frac{Wi}{Re}$$

where $V_{m}$, $D_{h}$, η, and ${\dot{\gamma }}_{c}$ indicate the mean flow velocity, the hydraulic diameter of the channel, the characteristic viscosity of the solution, and the characteristic shear rate, respectively.

As shown at B in Figure 1, cells with a blockage ratio (β=a/H, *H* is the microchannel height) larger than a threshold value are migrated laterally across the boundary from the viscoelastic fluid to the Newtonian fluid. In the present study, we determined this threshold value as the critical blockage ratio for effective cell washing. Particles/cells with a blockage ratio lower than the critical blockage ratio cannot be driven to the Newtonian fluid; therefore, they remain within the viscoelastic fluid, which can be used for the visualization of the viscoelastic fluid stream. Therefore, continuous on-chip washing of cells was achieved (C in Figure 1).

### 3.2. Effect of Viscoelasticity on Lateral Migration of Particles

To examine the effect of viscoelasticity on the flow characteristics of 13 μm fluorescent particles, the distributions of particles suspended in 50, 100, 250, and 500 ppm XG solutions were observed. The flow rate ratio of the sample (particle-containing XG solution) and sheath (PBS) flows was modulated to maintain a uniformly focused width of the sample in the inlet region, regardless of the sample viscosity. As the concentration of the XG solution increases, the viscosity increases (Table 1), and the focused width of the sample solution becomes wider owing to the viscosity difference between the sample and the sheath fluid [41,42,43]. The cells flowing at the center of the broadened sample stream cannot escape from the viscoelastic sample fluid and migrate across the boundary to the Newtonian sheath fluid. Therefore, to maintain a constant focused width ratio of 1:19 (sample to sheath) at the inlet region, the flow rate ratios of the sample and sheath fluids are required to be adjusted. For 50 and 100 ppm XG solution, the flow rate ratio was fixed at 1:19 with flow rates of 5 μL/min for sample (*Q*_sample_) and 95 μL/min for sheath (*Q*_sheath_), while the flow rate ratios were modulated to 1:39 and 1:99 for 250 ppm and 500 ppm XG solution, respectively, at the fixed total flow rate (100 μL/min).

**Figure 2:**
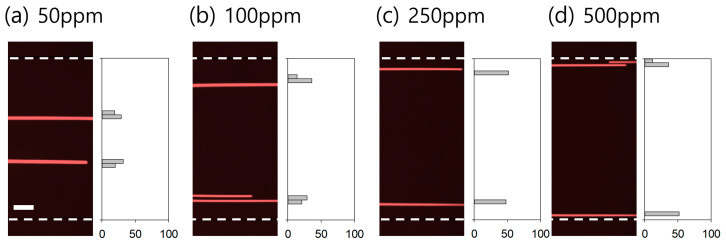
Effect of xanthan gum (XG) concentrations of (**a**) 50 ppm, (**b**) 100 ppm, (**c**) 250 ppm, and (**d**) 500 ppm on medium exchange of 13 μm particles from XG solution to PBS in the co-flow device. The total flow rates were 100 μL/min, and the focused width ratio of the sample to sheath flows was modulated as 1:19 for 50 and 100 ppm XG solution, 1:39 for 250 ppm XG solution, and 1:99 for 500 ppm XG solution. White dotted lines show the channel walls, and the X and Y axes indicate the normalized number of particles of 0–100 and the width of the expansion region of 0–800 μm. The scale bar is 100 μm.

Figure 2 shows the stacked microscopic images and normalized particle distribution of 13 μm particles. Particle distribution was examined in the expansion region (width 800 μm) at the outlet, which was for visualization of the flow streams of particles. The 800 μm expansion region was divided into 40 virtual bins, and the number of particles in each bin with 20 μm was normalized by the total number of particles flowing in the entire width.

At the inlet, 13 μm particles were focused at the channel center using a sheath fluid (PBS). Due to the rheological lift during the co-flow, many 13 μm particles were found to migrate laterally towards the channel walls even at a low concentration of 50 ppm (*Re* = 27.8, *Wi* = 510.1, *El* = 18.3) (Figure 2a). With an increase in concentration to 100 ppm (*Re* = 14.8, *Wi* = 616.7, *El* = 41.6), the fluorescent streams of 13 μm particles were located near the channel walls (Figure 2b). This indicated that most of the particles migrated laterally and were driven into the other medium (Newtonian fluid). The ratio of particles flowing near the channel walls increased with an increase in the concentration of the XG solution to 250 ppm (*Re* = 9.7, *Wi* = 1150, *El* = 119.0) and 500 ppm (*Re* = 5.6, *Wi* = 2086, *El* = 375.3), as shown in Figure 2c,d, owing to the enhanced shear thinning effect. To achieve effective medium exchange by fully utilizing the effect of rheological lift, which becomes stronger with increasing XG concentration, a sample solution with a high XG concentration, such as 500 ppm, is preferable. However, owing to its high viscosity, the device throughput is limited to ~1 μL/min.

Meanwhile, to validate that the lateral migration of 13 μm particles was enough for on-chip washing, the flow distributions of 500 nm particles in PBS and XG solutions at various concentrations were examined (see Supplementary Figure S1). Briefly, 500 nm particles suspended in PBS and XG solutions flowed with maintaining the focused width ratio at the inlet and showed no notable distribution difference in the width ratio at the inlet, which was due to the extremely small blockage ratio (*β* = 0.01). The distribution of 500 nm particles in PBS and 50 ppm XG solution showed a slight increase due to the effect of diffusion, since the diffusion coefficient increases with decreasing viscosity (0.88 mPa∙s for PBS and 0.8 mPa∙s for 50 ppm XG solution at room temperature). Then, the lateral migration of 13 μm particles in 100 ppm XG solutions shown in Figure 2b was regarded as being sufficient for medium exchange from a non-Newtonian to a Newtonian fluid. Therefore, although XG solutions with concentrations higher than 100 ppm can be used for particle/cell washing, the concentration of the XG solution was determined to be 100 ppm for further experiments, considering device throughput and lateral migration distance.

### 3.3. Determination of Critical Blockage Ratio for Efficient Washing

**Figure 3:**
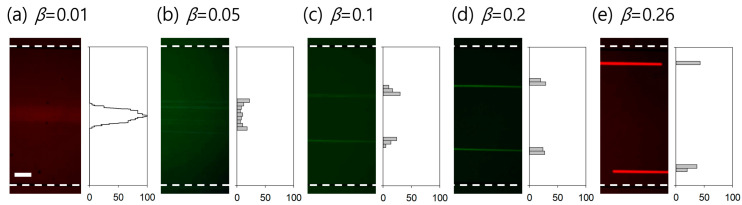
Effect of the blockage ratios of particles of (**a**) 0.01, (**b**) 0.05, (**c**) 0.1, (**d**) 0.2, and (**e**) 0.26 on medium exchange from 100 ppm XG solution to PBS in the co-flow device. White dotted lines show the channel walls. The scale bar denotes 100 μm.

To determine the threshold blockage ratio for efficient washing, fluorescent particles with diameters of 0.5, 2, 5, 10, and 13 μm were suspended in a 100 ppm XG solution, and the particle distribution was examined. The blockage ratios (*β*) of the particles were 0.01, 0.05, 0.1, 0.2, and 0.26, respectively. Figure 3 shows the particle size-dependent flow streams in the 100 ppm XG solution at fixed flow rates (*Q_sample_* 5 μL/min and *Q_sheath_* 95 μL/min). In Figure 3a, the Y-axis indicates a normalized fluorescent intensity of 0–100, while the Y-axes in Figure 3b–e denote the normalized number of particles flowing in each segment. For 500 nm particles (β=0.01), the particles flowed initially focused along the channel center at the inlet but with slight diffusion (Figure 3a), which was due to the small blockage ratio. Therefore, in the subsequent washing experiments, 500 nm fluorescent particles were used to visualize non-Newtonian fluid flow in the co-flow system. As shown in Figure 3b–e, as the particle size increased, the particles were driven further, and the equilibrium positions of the particles approached toward the channel walls. Particles with a blockage ratio higher than 0.1 started to show wall-directed lateral migration (Figure 3c). Using our experimental conditions (100 ppm XG solution, *Re* = 14.8, *Wi* = 616.7, *El* = 41.6), particles/cells with β≥0.1 can be medium-exchanged and washed to clean buffer for post-analysis. In this study, the target for cell washing, WBCs, has a size distribution of 11.0±5.0 μm (β=0.11±0.05) [20], so the lateral migration of WBCs will be sufficient for cell washing, based on the results in Figure 3d,e. In addition, although the length of the microchannel used in this study was fixed, multiple particle/cell separations and washing processes could be achieved using a co-flow system by modulating the channel length depending on the experimental objectives.

### 3.4. Clinical Application of Continuous On-Chip Washing

Washing WBCs in lysed blood samples has been demonstrated as a clinical application of continuous on-chip washing using a co-flow system. Isolation of WBCs from other blood components and buffer medium exchange are required for the proper treatment of various infectious diseases by the extraction of virological markers in WBCs [44,45] and for the detection of various diseases such as leukemia and human immunodeficiency virus infections [46]. Microfluidic approaches can address the bottlenecks of conventional methods (centrifugation and flow cytometry), such as the requirement of large volumes of samples and reagents, bulk setup, and trained personnel, which limits access in low-resource settings.

**Figure 4:**
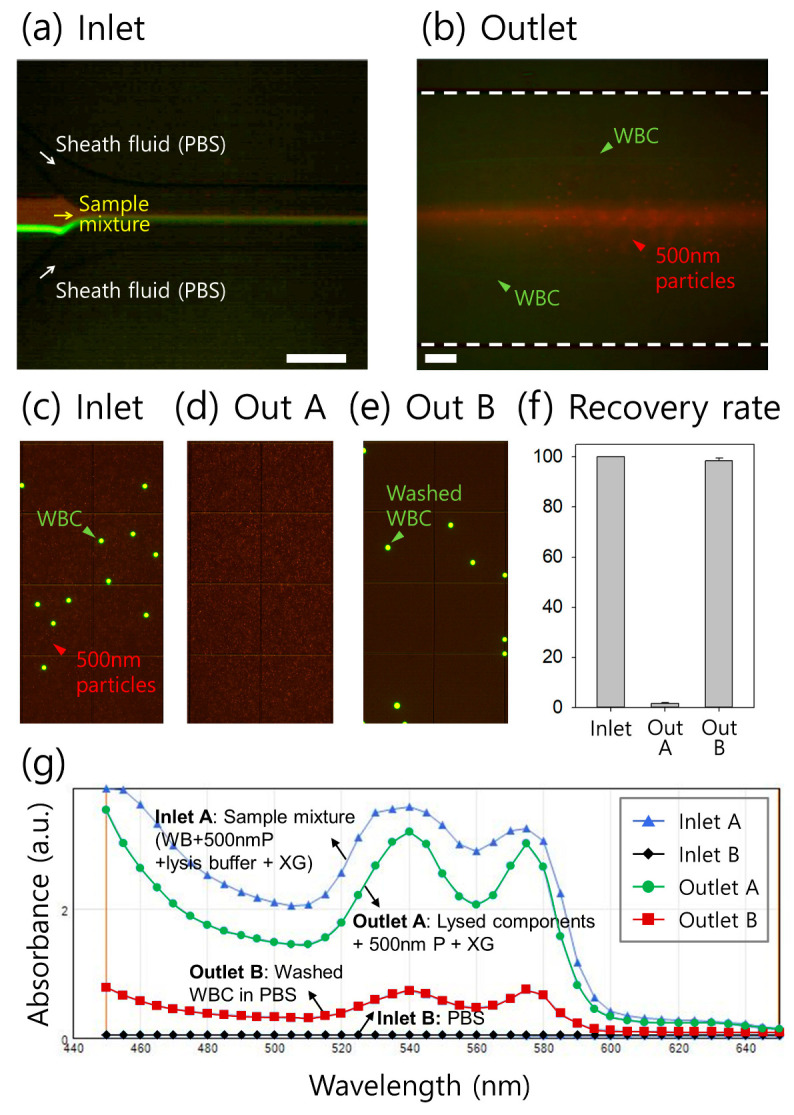
Application of the viscoelastic medium exchange system for white blood cell (WBC) washing at a total flow rate of 100 μL/min at the (**a**) inlet and (**b**) outlet expansion regions. Fluorescent images of the sample (**c**) before the washing process and after the washing process at the (**d**) outlet A and (**e**) outlet B. (**f**) Recovery rate based on quantitative analysis. (**g**) Absorbance spectra of the sample before and after the washing process.

Figure 4a,b show the stacked microscopic images at the inlet and outlet of the microchannel, respectively. Fluorescence-stained WBCs and 500 nm particles were initially focused by sheath flows injected from the side inlets (Figure 4a). At the outlet, WBCs were laterally driven toward the channel walls, while 500 nm particles visualizing the flow of the non-Newtonian fluid (XG solution) remained along the central region of the channel (Figure 4b)). Figure 4c–e shows the fluorescent images of the samples before and after the washing process. At the inlet, a mixture of WBCs and 500 nm particles was used (Figure 4c). After the washing process, migrated WBCs were collected in PBS at the side outlets (outlet B, Figure 4e) without 500 nm particles flowing to the center outlet (outlet A, Figure 4d). For the quantitative evaluation of WBC washing, the number of WBCs in each collected sample in outlets A and B was counted using a hemocytometer. The recovery rate of WBCs at outlet B exceeded 98% under our experimental conditions (Figure 4f).

To evaluate the on-chip washing performance of the WBCs in the XG solution further qualitatively, the absorbance spectra of the samples at the two inlets and two outlets were tested using scanning spectrophotometry (Figure 4g). PBS at inlet B had minimal absorbance without any peaks within the wavelength range of 440–650 nm, while the sample at inlet A containing lysed blood and fluorescent nanoparticles (500 nm diameter) in XG solution showed two major peaks at wavelengths of ~540 and 575 nm. XG has no notable absorption peaks within the 440–650 nm wavelength range, and it seems that the declining trend in absorbance within the 440–500 nm wavelength might be due to XG [47]. An amount of 500 nm fluorescent particles were used to visualize the viscoelastic flow stream, which did not affect the absorption spectrum within the wavelength range for analysis. The sample at inlet A also contained 1× SYBR Green for WBC staining, which shows extremely low absorbance values within the 440–650 nm wavelength, so that it has no effect on the current spectra results [48]. The washed WBCs collected at outlet B also showed two peaks with considerably reduced absorbance. These two major peaks at 540 and 575 nm wavelengths and one valley at 560 nm wavelength are the absorption characteristics of hemoglobin that exist in lysed blood samples [49]. Meanwhile, the sample from outlet A displayed a slight decrease in absorbance compared to that of the initial sample at inlet A. This indicates that diffusion of the initial sample into a Newtonian fluid (PBS) may have occurred during the on-chip washing process. Moreover, because of the large molecular size of xanthan gum, the molecular size ranges from hundreds to thousands of nanometers. Considering 100-nm-sized molecules, the mean square distance (*x*) that a molecule has diffused in time *t* was calculated as 1.24 μm:

(6) $$\langle x^{2}\rangle =6Dt$$

(7) $$D=\frac{KT}{3\pi \mu a}$$

where *D* is the diffusion coefficient and *K* is Boltzmann’s constant ($K=1.3806488\times 10^{-23}J/K$), respectively. The calculated distance (x=1.24 μm) indicates that the diffusion in the co-flow system can be negligible. Therefore, based on the absorbance spectra and theoretical analysis, efficient on-chip washing of WBCs from lysed blood to clean buffer (PBS) can be achieved using our XG solution-based co-flow system.

Our viscoelastic particle/cell washing device enables continuous on-chip washing. However, for the practical implementation of our device and the prospective replacement of centrifugation, its throughput is required to be improved. Device throughput can be further enhanced by decreasing the flow resistance, which means adopting multiple channels in a radial arrangement or device stacking in multiple layers [50,51,52]. Also, the aspect ratio of the microchannel can be modulated to have a wider channel by fabricating the fluidic device in a rigid thermoplastic resin [53], which facilitates the mass production of devices for commercialization. In addition, for further optimization, the effects of the total flow rate, channel length, and design of the outlet trifurcation are required to be considered to improve the washing performance.

## 4. Conclusions

In summary, we demonstrated continuous on-chip washing of particles/cells using a co-flow system of viscoelastic and Newtonian fluids, enabling the washing of WBCs in lysed blood samples. The proposed method utilizes an XG solution as a viscoelastic fluid, which has not been used in microfluidic co-flow systems. The effects of XG solution concentration and particle size were investigated to optimize the experimental parameters for particle/cell washing using the co-flow system. Therefore, 100 ppm XG solution was used for further experiments, and the critical particle blockage ratio for continuous washing was determined to be 0.1. In addition, 500 nm fluorescent particles were used to evaluate medium exchange over a viscoelastic/Newtonian fluid interface. Finally, WBCs were successfully washed from lysed blood samples by adding XG solution to clear buffer with a negligible diffusion effect, which was verified by absorbance spectra. Therefore, our co-flow system is a potential alternative to the conventional medium exchange and washing processes using centrifugation, allowing various biomedical applications.

## Figures and Tables

**Table 1:** Summary of measured rheological properties of XG solutions at 50, 100, 250, and 500 ppm.

XG Concentration (ppm)	$\eta_{0}$(mPa∙s) ^1^	$\eta_{\infty }$(mPa∙s) ^2^	*λ* (ms)
50	478	0.95	76.5
100	698	1.5	92.5
250	790	2.3	172.5
500	892	4	313

^1^ Zero-shear-rate viscosity; ^2^ infinite-shear-rate viscosity.

## Data Availability

Not applicable.

## Supplementary Materials

The following supporting information can be downloaded at: https://www.mdpi.com/article/10.3390/mi14091658/s1, Figure S1. Flow distribution of 500 nm particles in (a) PBS and XG solutions at various concentrations of (b) 50 ppm, (c) 100 ppm, (d) 250 ppm, and (e) 500 ppm.

## References

[1] Dineva M.A., Mahilum-Tapay L., Lee H. (2007). Sample preparation: A challenge in the development of point-of-care nucleic acid-based assays for resource-limited settings. Analyst.

[2] Duda D.G., Cohen K.S., Scadden D.T., Jain R.K. (2007). A protocol for phenotypic detection and enumeration of circulating endothelial cells and circulating progenitor cells in human blood. Nat. Protoc..

[3] Bacsa B., Kappe C.O. (2007). Rapid solid-phase synthesis of a calmodulin-binding peptide using controlled microwave irradiation. Nat. Protoc..

[4] Park S., Zhang Y., Wang T.-H., Yang S. (2011). Continuous dielectrophoretic bacterial separation and concentration from physiological media of high conductivity. Lab Chip.

[5] Tornay R., Braschler T., Demierre N., Steitz B., Finka A., Hofmann H., Hubbell J.A., Renaud P. (2008). Dielectrophoresis-based particle exchanger for the manipulation and surface functionalization of particles. Lab Chip.

[6] Nilsson A., Petersson F., Jönsson H., Laurell T. (2004). Acoustic control of suspended particles in micro fluidic chips. Lab Chip.

[7] Petersson F., Nilsson A., Jönsson H., Laurell T. (2005). Carrier Medium Exchange through Ultrasonic Particle Switching in Microfluidic Channels. Anal. Chem..

[8] Augustsson P., Persson J., Ekström S., Ohlin M., Laurell T. (2009). Decomplexing biofluids using microchip based acoustophoresis. Lab Chip.

[9] Augustsson P., Åberg L.B., Swärd-Nilsson A.-M.K., Laurell T. (2009). Buffer medium exchange in continuous cell and particle streams using ultrasonic standing wave focusing. Microchim. Acta.

[10] Peyman S.A., Iles A., Pamme N. (2008). Rapid on-chip multi-step (bio)chemical procedures in continuous flow—Manoeuvring particles through co-laminar reagent streams. Chem. Commun..

[11] Peyman S.A., Iles A., Pamme N. (2009). Mobile magnetic particles as solid-supports for rapid surface-based bioanalysis in continuous flow. Lab Chip.

[12] Morton K.J., Loutherback K., Inglis D.W., Tsui O.K., Sturm J.C., Chou S.Y., Austin R.H. (2008). Crossing microfluidic streamlines to lyse, label and wash cells. Lab Chip.

[13] Gossett D.R., Tse H.T.K., Dudani J.S., Goda K., Woods T.A., Graves S.W., Di Carlo D. (2012). Inertial Manipulation and Transfer of Microparticles Across Laminar Fluid Streams. Small.

[14] Shi X., Tan W., Lu Y., Cao W., Zhu G. (2021). A needle tip CCEA microfluidic device based on enhanced Dean flow for cell washing. Microsystems Nanoeng..

[15] Bogseth A., Zhou J., Papautsky I. (2020). Evaluation of Performance and Tunability of a Co-Flow Inertial Microfluidic Device. Micromachines.

[16] D’avino G., Maffettone P., Greco F., Hulsen M. (2010). Viscoelasticity-induced migration of a rigid sphere in confined shear flow. J. Non-Newton. Fluid Mech..

[17] Leshansky A.M., Bransky A., Korin N., Dinnar U. (2007). Tunable nonlinear viscoelastic “focusing” in a microfluidic device. Phys. Rev. Lett..

[18] Nam J., Lim H., Kim D., Jung H., Shin S. (2012). Continuous separation of microparticles in a microfluidic channel via the elasto-inertial effect of non-Newtonian fluid. Lab Chip.

[19] Nam J., Namgung B., Lim C.T., Bae J.-E., Leo H.L., Cho K.S., Kim S. (2015). Microfluidic device for sheathless particle focusing and separation using a viscoelastic fluid. J. Chromatogr. A.

[20] Lim H., Back S.M., Hwang M.H., Lee D.-H., Choi H., Nam J. (2019). Sheathless High-Throughput Circulating Tumor Cell Separation Using Viscoelastic non-Newtonian Fluid. Micromachines.

[21] Nam J., Jang W.S., Lim C.S. (2019). Non-electrical powered continuous cell concentration for enumeration of residual white blood cells in WBC-depleted blood using a viscoelastic fluid. Talanta.

[22] Kim J., Lim H., Jee H., Choo S., Yang M., Park S., Lee K., Park H., Lim C., Nam J. (2021). High-Throughput Cell Concentration Using A Piezoelectric Pump in Closed-Loop Viscoelastic Microfluidics. Micromachines.

[23] Choo S., Lim H., Kim T.E., Park J., Park K.B., Park C., Lim C.S., Nam J. (2022). A Continuous Microfluidic Concentrator for High-Sensitivity Detection of Bacteria in Water Sources. Micromachines.

[24] Lim H., Kim J.Y., Choo S., Lee C., Han B.J., Lim C.S., Nam J. (2023). Separation and Washing of Candida Cells from White Blood Cells Using Viscoelastic Microfluidics. Micromachines.

[25] Ha B., Park J., Destgeer G., Jung J.H., Sung H.J. (2016). Transfer of Microparticles across Laminar Streams from Non-Newtonian to Newtonian Fluid. Anal. Chem..

[26] Yuan D., Tan S.H., Sluyter R., Zhao Q., Yan S., Nguyen N.T., Guo J., Zhang J., Li W. (2017). On-Chip Microparticle and Cell Washing Using Coflow of Viscoelastic Fluid and Newtonian Fluid. Anal. Chem..

[27] Kang K., Lee S.S., Hyun K., Lee S.J., Kim J.M. (2013). DNA-based highly tunable particle focuser. Nat. Commun..

[28] Liu C., Zhao J., Tian F., Chang J., Zhang W., Sun J. (2019). λ-DNA- and Aptamer-Mediated Sorting and Analysis of Extracellular Vesicles. J. Am. Chem. Soc..

[29] Petri D.F.S. (2015). Xanthan gum: A versatile biopolymer for biomedical and technological applications. J. Appl. Polym. Sci..

[30] Kumar A., Rao K.M., Han S.S. (2018). Application of xanthan gum as polysaccharide in tissue engineering: A review. Carbohydr. Polym..

[31] Garcıa-Ochoa F., Santos V.E., Casas J.A., Gómez E. (2000). Xanthan gum: Production, recovery, and properties. Biotechnol. Adv..

[32] Hua D., Gao S., Zhang M., Ma W., Huang C. (2020). A novel xanthan gum-based conductive hydrogel with excellent mechanical, biocompatible, and self-healing performances. Carbohydr. Polym..

[33] Piola B., Sabbatini M., Gino S., Invernizzi M., Renò F. (2022). 3D Bioprinting of Gelatin–Xanthan Gum Composite Hydrogels for Growth of Human Skin Cells. Int. J. Mol. Sci..

[34] Li D., Xuan X. (2018). Fluid rheological effects on particle migration in a straight rectangular microchannel. Microfluid. Nanofluidics.

[35] Li D., Xuan X. (2019). The motion of rigid particles in the Poiseuille flow of pseudoplastic fluids through straight rectangular microchannels. Microfluid. Nanofluidics.

[36] Li D., Shao X., Bostwick J.B., Xuan X. (2019). Particle separation in xanthan gum solutions. Microfluid. Nanofluidics.

[37] Shankaran H., Alexandridis P., Neelamegham S. (2003). Aspects of hydrodynamic shear regulating shear-induced platelet activation and self-association of von Willebrand factor in suspension. Blood.

[38] Ho B.P., Leal L.G. (1974). Inertial migration of rigid spheres in two-dimensional unidirectional flows. J. Fluid Mech..

[39] Li D., Lu X., Xuan X. (2016). Viscoelastic Separation of Particles by Size in Straight Rectangular Microchannels: A Parametric Study for a Refined Understanding. Anal. Chem..

[40] Liu C., Xue C., Chen X., Shan L., Tian Y., Hu G. (2015). Size-Based Separation of Particles and Cells Utilizing Viscoelastic Effects in Straight Microchannels. Anal. Chem..

[41] Kang D., Song J.M., Yeom E. (2019). Design of microfluidic viscometer based on pressure estimation. J. Vis..

[42] Choi S., Park J.-K. (2010). Microfluidic Rheometer for Characterization of Protein Unfolding and Aggregation in Microflows. Small.

[43] Kim S., Kim K.C., Yeom E. (2018). Microfluidic method for measuring viscosity using images from smartphone. Opt. Lasers Eng..

[44] Choi J., Hyun J.-C., Yang S. (2015). On-chip Extraction of Intracellular Molecules in White Blood Cells from Whole Blood. Sci. Rep..

[45] Siliciano J.D., Kajdas J., Finzi D., Quinn T.C., Chadwick K., Margolick J.B., Kovacs C., Gange S., Siliciano R.F. (2003). Long-term follow-up studies confirm the stability of the latent reservoir for HIV-1 in resting CD^4+^ T cells. Nat. Med..

[46] Laxmi V., Joshi S.S., Agrawal A. (2022). Extracting white blood cells from blood on microfluidics platform: A review of isolation techniques and working mechanisms. J. Micromech. Microeng..

[47] Singh J., Dhaliwal A.S. (2020). Water retention and controlled release of KCl by using microwave-assisted green synthesis of xanthan gum-cl-poly (acrylic acid)/AgNPs hydrogel nanocomposite. Polym. Bull..

[48] Bruijns B., Tiggelaar R., Gardeniers H. (2017). Dataset of the absorption, emission and excitation spectra and fluorescence intensity graphs of fluorescent cyanine dyes for the quantification of low amounts of dsDNA. Data Brief.

[49] Liu P., Zhu Z., Zeng C., Nie G. (2012). Specific absorption spectra of hemoglobin at different PO2 levels: Potential noninvasive method to detect PO2 in tissues. J. Biomed. Opt..

[50] Hou H.W., Gan H.Y., Bhagat A.A.S., Li L.D., Lim C.T., Han J. (2012). A microfluidics approach towards high-throughput pathogen removal from blood using margination. Biomicrofluidics.

[51] Khoo B.L., Warkiani M.E., Tan D.S.-W., Bhagat A.A.S., Irwin D., Lau D.P., Lim A.S.T., Lim K.H., Krisna S.S., Lim W.-T. (2014). Clinical Validation of an Ultra High-Throughput Spiral Microfluidics for the Detection and Enrichment of Viable Circulating Tumor Cells. PLoS ONE.

[52] Warkiani M.E., Khoo B.L., Tan D.S.-W., Bhagat A.A.S., Lim W.-T., Yap Y.S., Lee S.C., Soo R.A., Han J., Lim C.T. (2014). An ultra-high-throughput spiral microfluidic biochip for the enrichment of circulating tumor cells. Analyst.

[53] Hupert M.L., Jackson J.M., Wang H., Witek M.A., Kamande J., Milowsky M.I., Whang Y.E., Soper S.A. (2014). Arrays of high-aspect ratio microchannels for high-throughput isolation of circulating tumor cells (CTCs). Microsyst. Technol..

