# Supplementary material for: Continuous On-Chip Cell Washing Using Viscoelastic Microfluidics
Source: Micromachines (Basel). 2023 Aug 25;14(9):1658. doi: 10.3390/mi14091658 (PMC10535438; doi:10.3390/mi14091658)
Supplement: Supplementary file 1 [file micromachines-14-01658-s001.zip › micromachines-2558693-supplementary.pdf]

micromachines

MDPI

Supplementary Materials

Continuous on-Chip Cell Washing Using Viscoelastic Microfluidics

Hyunjung Lim [1,†], Minji Kim [2,†], Yeongmu Kim [3], Seunghee Choo [4], Tae Eun Kim [3], Jaesung Han [5], Byoung Joe Han [6], Chae Seung Lim [7,*] and Jeonghun Nam [3,6,*]

1 Interdisciplinary Program in Precision Public Health (PPH), Korea University, Seoul 02841, Republic of Korea; hyunjunglim.email@gmail.com
2 Department of AI Electrical and Electronic Engineering, Incheon Jaeneung University, Incheon 22573, Republic of Korea; minjizzang73@gmail.com
3 Artificial Intelligence(AI)-Bio Research Center, Incheon Jaeneung University, Incheon 21987, Republic of Korea
4 College of Life Sciences and Bio Engineering, Incheon National University, Incheon 22012, Republic of Korea
5 Department of Mechanical and control technologies, Seoul Cyber University, Seoul 01133, Republic of Korea
6 Department of Digital Biotech, Incheon Jaeneung University, 22573, Incheon, Republic of Korea
7 Department of Laboratory Medicine, College of Medicine, Korea University, Seoul 08307, Republic of Korea
* Correspondence: malarim@korea.ac.kr (C.S.L.); jhnam77@gmail.com (J.N.); Tel.: +82-2-2626-1514 (C.S.L.); +82-32-890-7515 (J.N.).
† These authors contributed equally to this work.

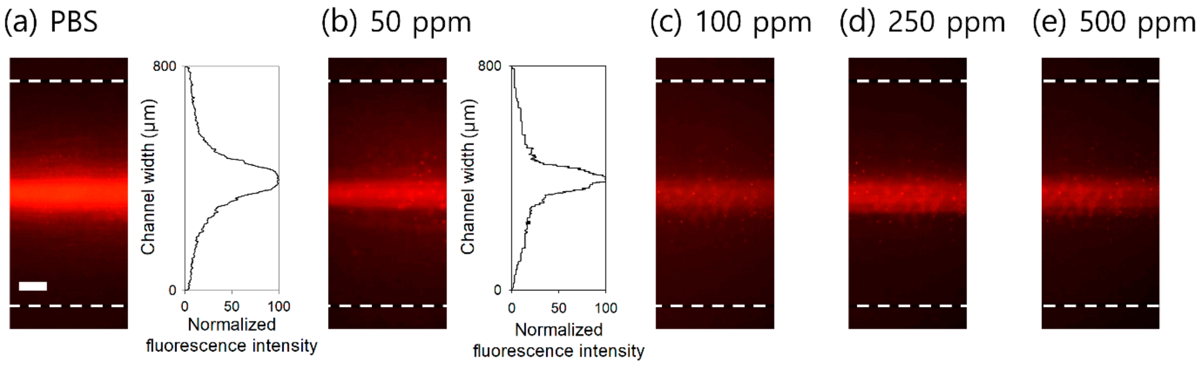

Figure S1. Flow distribution of 500 nm particles in (**a**) PBS and XG solutions at various concentrations of (**b**) 50 ppm, (**c**) 100 ppm, (**d**) 250 ppm, and (**e**) 500ppm, respectively. White dotted lines show the channel walls and the X and Y axes indicate the normalized number of particles of 0-100 and the width of the expansion region of 0-800 μm. The scale bar is 100 μm.
